# Supplementary material for: Hyperinflammatory Immune Response in COVID-19: Host Genetic Factors in Pyrin Inflammasome and Immunity to Virus in a Spanish Population from Majorca Island
Source: Biomedicines. 2023 Sep 16;11(9):2548. doi: 10.3390/biomedicines11092548 (PMC10525993; doi:10.3390/biomedicines11092548)
Supplement: Supplementary file 1 [file biomedicines-11-02548-s001.zip › biomedicines-2544670-supplementary.pdf]

Patients	Age	Sex	Severe/ Critical	Comorbidities				Laboratory data														Genetic risk markers		
				Obesity	Dyslipemia	Diabetes	Hypertension	Ferritin (ng/mL)	D dimer (ng/mL)	LDH (U/L)	AST (U/L)	Lymph. counts (x 10^3/uL)	NLR	Cytopenias	IL-6	IL-10	IL-1 β	IL-1Ra	IL-18	sCD25	*MEFV* AA/AA	*IFNAR2* p.Phe8Ser	*CARMIL2* p.Val181Met
HP-1	61	M	3	No	No	Yes	Yes	2109	2332	434	20	0,25	12,5	L, A, T	N.A	N.A	N.A	N.A	N.A	N.A	2	0	0
HP-2	70	M	3	No	No	Yes	Yes	3801	3588	713	48	0,3	15,3	L, A	N.A	N.A	N.A	N.A	N.A	N.A	1	0	0
HP-3	63	M	3	No	Yes	Yes	Yes	435	3330	553	42	0,38	15,9	L, A	5,6	25,2	5,2	24,2	35,1	892	2	0	0
HP-4	56	M	3	Yes	No	Yes	No	1107	3398	412	98	0,59	10,9	L, A, T	N.A	N.A	N.A	N.A	N.A	N.A	2	0	0
HP-5	53	M	3	Yes	Yes	No	No	2872	2821	528	108	0,31	29,7	L, A	1149	21	0,6	40,5	47,6	N.A	0	0	0
HP-6	65	M	3	No	Yes	No	Yes	2540	2034	359	85	0,2	43,0	L, A, T	89,4	63,1	8,3	15,3	19,7	2886	0	1	0
HP-7	65	M	2	No	No	No	No	2731	679	381	40	0,81	15,4	L, A	175	46,5	1,7	58,4	73,5	746	2	0	0
HP-8	64	M	3	No	No	No	No	3168	16269	410	174	0,53	16,8	L, A	189	81,5	23,5	35,2	120	1167	2	0	0
HP-9	65	F	3	No	No	No	Yes	1683	647	532	78	0,34	6,4	L, A, N	43,5	13	12,8	68,7	47	N.A	0	2	0
HP-10	66	M	2	No	No	No	No	6411	3231	433	69	0,18	5,3	L, A, N, T	N.A	N.A	N.A	N.A	N.A	N.A	0	1	0
HP-11	78	F	3	No	Yes	No	No	2732	3525	645	262	0,32	26,3	L, A, T	156,0	96,4	6,6	170,1	106,5	N.A	1	0	0
HP-12	70	M	3	No	Yes	Yes	Yes	5965	3259	459	286	0,67	3,0	L, A, T	N.A	N.A	N.A	N.A	N.A	N.A	0	0	0
HP-13	65	M	3	Yes	Yes	Yes	Yes	2005	2161	517	221	0,57	8,9	L, A	248,0	108,9	58,9	251,8	85,5	N.A	1	0	0
HP-14	52	M	2	Yes	No	No	No	345	274	371	34	0,25	58,0	L, A, T	259,0	51	148,6	236,2	189,6	476	1	0	0
HP-15	58	M	3	No	No	No	No	1995	2426	499	112	0,17	30,8	L, A	60,0	33	< 1,6	29,9	29,6	1338	2	1	1
HP-16	66	M	3	Yes	Yes	Yes	Yes	3844	2991	602	88	0,63	20,2	L, A	49	22,9	19,5	147,3	49,7	N.A	2	0	0
HP-17	48	M	2	Yes	Yes	No	No	1448	1849	402	71	0,74	6,4	L, A	N.A	N.A	N.A	N.A	N.A	N.A	2	0	0
HP-18	42	M	2	No	No	No	No	6198	321	458	99	0,75	8,2	L	158,8	42,9	28,1	333	125,7	N.A	0	0	0
HP-19	20	F	2	No	No	No	No	8825	3302	1075	N.A	0,29	40,0	L, T	N.A	N.A	N.A	N.A	N.A	N.A	0	1	0
HP-20	76	F	2	No	No	Yes	Yes	1088	1623	542	66	1,8	6,0	A, T	22,5	11,3	2,7	49,4	17	N.A	1	0	1
HP-21	58	M	2	No	No	No	No	11872	445	514	52	0,69	5,4	L, A	149	21,5	14,7	143,6	14,4	N.A	1	0	0
HP-22	19	M	2	No	No	No	No	515	2746	491	47	0,49	29,6	L	88	71,5	3,8	379,7	39,6	579	0	0	0
HP-23	73	M	2	No	Yes	No	Yes	125542	3204	2435	905	0,2	94,0	L, A, T	21,6	1,8	4,9	43,7	1,2	814	1	1	0
HP-24	51	F	3	Yes	No	No	No	1921	451	503	115	0,8	18,5	L	32	11,1	6,6	90,6	94,6	N.A	1	1	0
HP-25	55	M	2	No	Yes	Yes	Yes	2290	2503	705	62	0,42	11,9	L, A	33,4	11,6	3,1	64,9	16,1	N.A	2	0	1
HP-26	66	M	2	No	Yes	Yes	Yes	2432	797	1087	94	0,49	26,9	L, A	N.A	N.A	N.A	N.A	N.A	N.A	2	0	0
HP-27	67	M	3	No	Yes	No	No	1671	2727	394	76	0,49	18,5	L	N.A	N.A	N.A	N.A	N.A	N.A	1	1	0
HP-28	59	F	2	No	Yes	No	No	1308	2025	674	78	0,42	1,4	L, A, N T	N.A	N.A	N.A	N.A	N.A	N.A	1	1	0
HP-29	57	F	3	No	Yes	No	No	605	508	601	60	0,43	17,1	L	2213	79,5	18,3	602,6	188,5	N.A	2	1	0
HP-30	49	M	3	Yes	Yes	No	No	2860	1934	598	47	0,43	52,3	L, A	N.A	N.A	N.A	N.A	N.A	N.A	1	0	0
HP-31	48	M	3	No	Yes	No	No	3905	1043	603	52	0,49	16,8	L	85,0	76,4	3,1	176	26,2	488	1	0	0
HP-32	70	M	2	Yes	No	No	No	11674	1593	404	59	0,51	11,5	L, A, T	31	1,8	13,1	27,9	19,9	N.A	2	1	0
HP-33	32	M	3	Yes	Yes	Yes	Yes	3688	3524	611	64	0,56	10,7	L	N.A	N.A	N.A	N.A	N.A	N.A	1	0	1
HP-34	63	M	3	No	Yes	No	Yes	6122	1401	609	131	0,44	4,3	L, T	1829	86,2	4,9	2578,9	103,4	N.A	2	0	0
HP-35	57	M	2	No	Yes	No	No	4293	651	331	98	0,64	18,0	L	N.A	N.A	N.A	N.A	N.A	N.A	0	0	0
HP-36	73	M	2	No	No	No	Yes	2008	501	408	74	0,46	20,1	L, A	N.A	N.A	N.A	N.A	N.A	N.A	2	1	0
HP-37	69	M	2	No	No	No	No	4063	3595	405	70	0,29	6,9	L, A	N.A	N.A	N.A	N.A	N.A	N.A	2	0	0
HP-38	54	M	2	Yes	Yes	No	Yes	2598	825	393	55	0,49	14,0	L, A	N.A	N.A	N.A	N.A	N.A	N.A	0	0	1
HP-39	69	M	3	No	no	No	No	567	3377	600	54	N.A	N.A	A	2100	479,9	431	451,9	329	1327	2	0	1
HP-40	75	M	2	No	No	No	Yes	1190	251	305	66	1,05	6,1	Low E	N.A	N.A	N.A	N.A	N.A	N.A	2	0	0
HP-41	59	F	3	No	No	No	No	2235	749	505	31	0,67	9,4	L, A, Low E	N.A	N.A	N.A	N.A	N.A	N.A	1	1	0
HP-42	61	F	2	Yes	Yes	Yes	Yes	1103	2142	405	50	0,8	14,5	L, Low E	N.A	N.A	N.A	N.A	N.A	N.A	0	0	0
HP-43	54	M	2	No	Yes	No	No	1280	156	291	42	0,87	8,8	L, Low E	N.A	N.A	N.A	N.A	N.A	N.A	0	0	1
HP-44	59	M	2	No	No	No	No	2487	8329	471	42	0,58	8,4	L, A, Low E	N.A	N.A	N.A	N.A	N.A	N.A	2	0	0
HP-45	80	F	2	No	Yes	Yes	Yes	1957	253	558	48	0,87	12,3	L, A, Low E	N.A	N.A	N.A	N.A	N.A	N.A	1	0	0
HP-46	54	M	2	Yes	Yes	No	No	2197	521	318	71	0,64	10,7	L, Low E	N.A	N.A	N.A	N.A	N.A	N.A	0	0	0
HP-47	61	M	2	No	NO	NO	Yes	4012	108	357	25	0,61	4,0	L, Low E	N.A	N.A	N.A	N.A	N.A	N.A	1	0	0
HP-48	70	M	2	No	Yes	No	Yes	388	829	296	67	0,68	28,5	L, A, Low E	N.A	N.A	N.A	N.A	N.A	N.A	1	0	0
HP-49	66	M	2	No	Yes	Yes	Yes	698	483	354	42	0,35	17,9	L	N.A	N.A	N.A	N.A	N.A	N.A	0	0	0
HP-50	55	M	2	No	No	Yes	No	1837	284	499	44	0,65	5,8	L, Low E	N.A	N.A	N.A	N.A	N.A	N.A	2	1	0
HP-51	68	F	2	Yes	Yes	NO	No	601	479	329	95	1,09	5,3	A, Low E	N.A	N.A	N.A	N.A	N.A	N.A	1	0	0
HP-52	59	F	2	Yes	Yes	NO	No	1308	2025	674	78	0,42	1,2	L, T, N, A	N.A	N.A	N.A	N.A	N.A	N.A	1	1	0

Table S1. Age, sex, Symptomatology classification, comorbidities, laboratory data and genetic risk markers of Hyperinflammatory patients. Current age (years) and gender (M: male, F: female). Symptomatology classification (2: Severe or 3: Critical COVID-19 infection). Presence (Yes) or absence (No) of the following comorbidities: Obesity (Body mass index >30), dyslipemia, diabetes and hypertension. Laboratory data included serum levels of ferritin, D-dimer, Lactate dehydrogenase (LDH) and Aspartate aminotransferase (AST). Absolute lymphocyte (Lymph) counts, Neutrophil-lymphocyte ratio (NLR), Cytopenias (L: lymphopenia, A: anemia, T: thrombocytopenia, N: neutropenia, Low E: low eosinophil counts). Serum cytokines levels of IL-6, IL-10, IL-1**β**, IL-1Ra, IL-18 and soluble interleukin 2 receptor (sCD25). Presence (1: variant in an heterozygous state, 2: variant in an homozygous state) or absence (0: not variant) of rs1231123A/A-rs1231122A/A *MEFV* haplotype (*MEFV* AA/AA) and p.Phe8Ser_*IFNAR2* and p.Val181Met_*CARMIL2* variants. N.A: Not available

PATIENTS	Age	Sex	Symptomatology	Comorbidities				Genetic risk markers		
				Obesity	Dyslipemia	Diabetes	Hypertension	*MEFV* AA/AA	*IFNAR2* p.Phe8Ser	*CARMIL2* p.Val181Met
AM-RISK-1	98	M	Asymptomatic	No	No	No	No	0	0	0
AM-RISK-2	94	F	Asymptomatic	No	Yes	Yes	Yes	1	0	0
AM-RISK-3	91	F	Asymptomatic	No	No	No	No	0	0	0
AM-RISK-4	85	M	Asymptomatic	No	No	No	No	1	1	0
AM-RISK-5	99	F	Asymptomatic	No	No	No	Yes	1	0	0
AM-RISK-6	90	F	Asymptomatic	No	Yes	No	Yes	2	0	0
AM-RISK-7	100	F	Asymptomatic	No	Yes	No	No	0	0	0
AM-RISK-8	91	F	Asymptomatic	No	No	No	No	0	0	0
AM-RISK-9	81	F	Asymptomatic	No	No	No	Yes	1	0	0
AM-RISK-10	89	F	Asymptomatic	No	No	No	Yes	1	0	0
AM-RISK-11	70	M	Asymptomatic	Yes	No	Yes	Yes	1	0	0
AM-RISK-12	88	M	Asymptomatic	No	No	Yes	Yes	1	0	0
AM-RISK-13	93	F	Asymptomatic	N.A	No	Yes	Yes	0	0	0
AM-RISK-14	98	F	Fatigue	N.A	No	No	Yes	1	0	0
AM-RISK-15	95	F	Asymptomatic	N.A	Yes	No	Yes	1	0	0
AM-RISK-16	81	F	Asymptomatic	N.A	No	No	No	0	0	0
AM-RISK-17	70	M	Fatigue	Yes	No	Yes	Yes	1	0	0
AM-RISK-18	78	F	Fatigue	N.A	No	Yes	Yes	0	0	0
AM-RISK-19	90	M	Fatigue	No	No	No	Yes	2	0	0
AM-RISK-20	81	F	Throat pain	N.A	No	Yes	No	0	0	0
AM-RISK-21	70	M	Fatigue	N.A	Yes	Yes	Yes	1	0	0
AM-RISK-22	90	F	Asymptomatic	Yes	Yes	No	Yes	0	0	0
AM-RISK-23	81	F	Fever/Fatigue	No	No	No	No	2	1	0
AM-RISK-24	85	M	Asymptomatic	No	No	No	No	1	0	0
AM-RISK-25	88	F	Asymptomatic	No	No	No	No	1	0	0
AM-RISK-26	85	F	Asymptomatic	No	No	No	Yes	2	0	0
AM-RISK-27	97	F	Asymptomatic	No	No	No	No	1	1	0
AM-RISK-28	81	F	Asymptomatic	Yes	No	No	Yes	1	0	0
AM-RISK-29	85	M	Asymptomatic	No	Yes	Yes	Yes	1	0	0
AM-RISK-30	86	F	Asymptomatic	No	No	No	Yes	1	0	0
AM-RISK-31	80	F	Asymptomatic	No	No	No	No	2	0	0
AM-RISK-32	78	M	Fever/Fatigue	No	No	No	Yes	1	0	0
AM-RISK-33	81	M	Asymptomatic	Yes	Yes	No	Yes	1	0	0
AM-RISK-34	88	F	Asymptomatic	Yes	No	No	Yes	1	0	0
AM-RISK-35	85	M	Asymptomatic	Yes	Yes	No	No	1	0	0
AM-RISK-36	81	F	Cough/ throat pain	N.A	No	No	Yes	1	0	0
AM-RISK-37	74	F	Throat pain/Fatigue	N.A	Yes	Yes	No	1	0	0
AM-RISK-38	89	F	Cough/ throat pain	N.A	No	No	No	1	0	0
AM-RISK-39	71	M	Fatigue	N.A	No	No	Yes	2	0	0
AM-RISK-40	75	M	Fever	N.A	No	No	Yes	1	0	0

Table S2. Age, sex, symptomatology, comorbidities and genetic risk markers of AM-Risk patients. Current age (years) and gender (M: male, F: female). Symptomatology associated with COVID-19 infection. Presence (Yes) or absence (No) of the following comorbidities: Obesity (Body mass index >30), dyslipemia, diabetes and hypertension. Presence (1: variant in an heterozygous state, 2: variant in an homozygous state) or absence (0: not variant) of rs1231123A/A-rs1231122 A/A *MEFV* haplotype (*MEFV* AA/AA), p.Phe8Ser_*IFNAR2* and p.Val181Met_*CARMIL2*. N.A: Not available.
